# The Influence of Top Management Team Human Capital on Sustainable Business Growth

**DOI:** 10.3389/fpsyg.2021.773689

**Published:** 2021-11-25

**Authors:** Yaoping Shen, Qian Zheng, Xinghui Lei, Fengpei Hu

**Affiliations:** ^1^School of Economics and Management, Tongji University, Shanghai, China; ^2^School of Management, Zhejiang University of Technology, Hangzhou, China

**Keywords:** decision-making quality, founder characteristics, human capital, sustainable business growth, top management team

## Abstract

Traditionally, enterprises have subscribed to the belief that top management team (TMT) human capital is of great influence to a specific entity. While long being a question of interest in the field of management, the extant literature rarely discusses the impact of TMT human capital on sustainable business growth. By examining data obtained from 535 TMT members of private enterprises in Zhejiang Province PRC, and investigate the mediating effect of decision-making quality as well as the moderating effect of founder characteristics, we analyze the positive operating mechanisms of TMT human capital on sustainable business growth. Interestingly, the single most striking observation to emerge from the empirical investigation was: (1) TMT human capital has a significant positive impact on sustainable business growth; (2) decision-making quality fully mediates the relationship between TMT human capital and sustainable business growth; and (3) the more open-minded the founders, the stronger the mediating effect of decision-making quality in the relationship between TMT human capital and sustainable business growth. This research has expanded the perspective and scope of the research on TMT human capital, and its practical usage is discussed.

## Key Points

- TMT human capital has a significant positive impact on sustainable business growth in enterprises.
- Decision-making quality fully mediates the relationship between TMT human capital and sustainable business growth.
- The more open-minded the founders, the stronger the mediating effect of decision-making quality in the relationship between TMT human capital and sustainable business growth.

## Introduction

The market environment faced by businesses today is complex, constantly evolving, and full of challenges. Individual abilities are inadequate to ensure the competitive advantage of companies. To operate business smoothly, business managers need to form teams comprising senior managers from different departments and fields, and to utilize their human capital effectively to facilitate collaboration in business operations and decision-making. In recent years, various social problems have arisen due to the negligence of senior management personnel. For instance, in 2011, the News of the World was found to be involved in illegal interception and eavesdropping on private telephone communications and was forced to shut down, leading the Murdoch Group to a phone-hacking scandal. Since 2017, when LeTV experienced a funding crisis, Chairman Yueting Jia has been gradually withdrawing himself from core management and turning toward the automotive industry; yet, no good news has emerged thus far. In 2018, Facebook’s breach of user data confidentiality led to serious violations of its commitment to protect user privacy and damaged the company’s reputation. The bicycle sharing company, OFO, has also been affected by internal management conflicts. All these incidents indicate that there are various obstacles to business development and that their occurrence is always closely related to the company’s senior management, who are responsible for making business decisions. When the management is lenient and neglects certain threats, enterprise development is inevitably affected.

Existing research confirms the importance of top management team (TMT) human capital for sustainable business growth (Patzelt et al., 2008; Nuscheler et al., 2018; Shen et al., 2020; Neffe et al., 2021). In this critical period of global economic slowdown and China’s social and economic transformation, strengthening strategic leadership among TMTs and the role of TMT human capital is the key to achieve operational efficiency and sustainable business growth. Therefore, clarifying the mechanisms by which TMT human capital affects sustainable business growth is a popular issue faced by both the academia and the industry. From a resource perspective, TMT human capital’s promotion of sustainable business growth is essentially a decision-making process that drives an enterprise’s resource acquisition and integration. In other words, TMT human capital entails the collaboration of value orientation and capability structures. Through the decision-making process, a company’s TMT needs to weigh costs and benefits to balance different types of resources. Several key factors would drive the role of TMT human capital. The institutional theory postulates that corporate reform is the process of replacing old systems with new ones. The demands and disputes arise from the reform process between new and old organizations form the main features of a corporate organizational context and the factors that influence the role of TMT human capital. A relatively lenient organizational atmosphere indicates that the TMT’s practical management is relatively simple, such that its main focus is on business development strategy. An atmosphere lacking leniency renders it difficult for the organization’s members to make decisions; thus, they passively execute instructions. Therefore, TMTs need to spend additional operational efforts to overcome the disadvantages resulting from this undesirable situation (Lohrke et al., 2004; Chemin, 2021; Han et al., 2021).

To a certain extent, TMTs also determine the decision-making ability and management capabilities of enterprises. They are the driving force for maintaining and promoting business development; TMTs’ decisions and management skills have a direct influence on the success or failure of enterprises. Venugopal et al. (2017) have found that TMT behavioral integration mediates the effect of TMT connectedness, and TMT cross-functional interfacing mechanisms on organizational ambidexterity, which in turn affects organizational decision making. Competition among corporations is ultimately centered on talent as talent reflects knowledge and information. The TMT is undoubtedly an important talent resource for enterprise creation and plays a vital role in the innovation and development of organizations. This is because a company’s TMT possesses the knowledge and skills that cannot be easily imitated, transferred, or completely replaced by competitors (i.e., human capital). The human capital owned by its senior management team is an important source of a company’s competitive advantage, and The highly complex knowledge held by members of TMTs is essential for a firm’s success (Chen et al., 2019; Roh et al., 2019). A well-functioning TMT can effectively balance the interests of stakeholders and focuses on achieving long-term organizational goals (Boivie et al., 2016). In other words, it is an important driver of a company’s competitive advantage. This is especially applicable to private enterprises, whose founder characteristics have been found to be a significant factor for enterprise growth and sustainability (Cock et al., 2020). However, Chinese private enterprises face certain problems related to the establishment of senior management teams, in that their teams have not been able to demonstrate their corporate management abilities fully. Therefore, under normal economic conditions, private enterprises that aim to grow in an increasingly fierce market environment continuously must build strong senior management teams, which is necessary for achieving sustainable growth.

Based on this background, this study investigates the TMTs of private enterprises in Zhejiang Province PRC, and discusses the influences of TMT human capital, founder characteristics, and decision-making quality on sustainable business growth. We focus on the demographic characteristics of founders, including gender, age, years of work experience, and the degree of differences between them. And we combine these variables with founder characteristics to explore their impact on sustainable business growth.

This study reveals the important influence mechanisms of TMT human capital on sustainable business growth, and tests the vital role of founder characteristics and decision-making quality in gaining competitive advantage in a business environment. Previous studies have confirmed that the decision-making quality of entrepreneurs and corporate executives has a corresponding impact on business performance (Liu et al., 2021); however, few studies discuss the influence mechanism in depth. As enterprises continue to grow, the decision-making quality of corporate executives drives their development, while the founders’ characteristics determine the level of decision-making quality (Acedo and Jones, 2007). This provides an important theoretical basis for this study. Accordingly, this study not only validates the mediating effect of decision-making quality on the relationship between TMT human capital and sustainable business growth, but also examines the importance of founder characteristics in moderating the relationship between TMT human capital and decision-making quality. Moreover, the study reveals that founder characteristics affect decision-making quality, thereby contributing to this area of research.

Most existing studies are based on relatively mature market systems, while the market environment in emerging markets (e.g., China) is far more complex. Currently, there is a small but growing strand of literature focusing on how TMT human capital and decision-making quality affect corporate growth in emerging markets. This study considers this social background in analyzing the effect of TMT human capital on sustainable business growth in China’s private enterprises. Thus, it provides theoretical support for the establishment and management of private enterprises in China. By introducing founder characteristics as a variable, we were able to develop a more comprehensive theoretical model to demonstrate the relationships between TMT human capital, decision-making quality, founder characteristics, and sustainable business growth.

The sample of this study covers private enterprises in Zhejiang Province, PRC. As such, the results serve as an important reference for the sustainable growth of private enterprises both at the regional and national levels. In an increasingly complex internal and external environment, executive team members must focus on improving their abilities and in turn, decision-making quality in order to achieve sustainable business growth. Business competition is not merely a competition of products or companies. It also involves the competition of the management and thinking models of the senior management team. When building a senior management team, it is vital to improve the competency of its members and to systematically examine whether they can make high-quality decisions to ensure sustainable growth of the company.

The rest of the paper unfolds as follows. In section “Literature Review and Hypothesis Development,” we review the related literature and develop hypotheses. Section “Sample and Methodology” describes the sample and methodology. Section “Results and Analysis” presents empirical results while section “Concluding Remarks” concludes.

## Literature Review and Hypothesis Development

In this section, we review the related literature and develop hypotheses.

### Top Management Team Human Capital and Sustainable Business Growth

Research on TMT stems from the “Upper Echelons Theory” proposed by Hambrick and Mason (1984). They define all managers at the executive team level as TMT, including vice presidents, vice presidents, presidents, etc. Lu and Ge (2009) point out that TMT is a combination of high knowledge, high intelligence and high collaboration. It does not only have the characteristics and functions of general teams, but also mainly relies on high-quality individual human capital. They divide TMT human capital into two levels. The narrow TMT human capital refers to TMT’s team human capital, while the broad TMT human capital includes both the team’s human capital and the individual human capital of TMT members.

Current research on TMT mainly focuses on the composition of senior managers and the distribution of characteristics among individual members within the team. Most studies focus on the links between demographic characteristics and changes, strategies, strategic shifts, managerial turnover, and organizational performance. Demographic characteristics of senior managers include age, tenure, expertise, and education. Researchers consider that these reflect the special experiences, values, and personalities of senior management personnel and affect the entire team.

Some studies suggest that the demographic heterogeneity of TMTs, including age, educational level, and tenure, is positively correlated to organizational performance (Ormiston et al., 2021); in other words, it has a positive effect on business growth (Smith et al., 1994). Patzelt et al. (2008) examine the data for 99 German biotechnology companies to demonstrate that the experience of TMTs has a positive influence on business management and operations. Furthermore, Yoo et al. (2009) argue that TMTs’ external communication and ties with associated and external enterprises influence corporate strategy choices and performance. Using data collected from a sample of enterprises, the authors find that the presence of industry associations facilitates effective communication among TMT members and improves company performance. Similarly, Su et al. (2017) use configuration solutions of the leader-TMT dynamics to illustrate distinct HR strategies to form a well-functioning TMT, and these are equally effective for achieving desired internationalization goals. Cannella et al. (2008) find that the diversity of TMTs has an inconsistent impact on business performance. The authors believe that internal and external environments are equally important for TMT effectiveness, and affect corporate performance. TMT capabilities are not merely an addition of individual abilities; rather, they need to be integrated through certain processes and mechanisms. Whether the combination of individual abilities is reasonable or not determines the overall effectiveness of TMT to a certain extent, which, in turn, determines the efficiency of business operations and affects the sustainable growth of enterprises. Based on the above, the following hypothesis is established.

*Hypothesis 1: TMT human capital has a significant and positive impact on sustainable business growth.*

### Mediating Effect of Decision-Making Quality

Information on organizational environment, personal preferences, and proposal evaluation methods are the key to effective decision-making. These are related to personal experience and understanding of problems, particularly complex problems. Numerous factors, including multiple objectives, uncertainty, temporal dynamics, and competitiveness, may make it impossible for individual abilities to cope with the problem. Thus, it is necessary to exercise collective wisdom and involve additional individuals in decision-making. Moreover, high-quality decision-making is a cycle of collective exploration and practice, including the continuous acquisition as well as the understanding, integration, and institutionalization of new knowledge and skills. To facilitate high-quality decisions, business leaders must commit and provide support, as they are responsible for creating the appropriate organizational environment for high decision-making quality. Georgakakis et al. (2017) find that the behavior of TMTs affects firm performance, and the possible internal mechanism is decision-making quality. Nielsen and Daniels (2012) further propose that the organization’s leaders must adopt the roles of learners and educators. When members of an organization consider their leaders to be fair teachers who share useful knowledge, they will take learning more seriously and will ensure successful organizational knowledge integration. Kogut and Ritov (2007) believe that the decision-making behavior of organizational managers not only impacts the culture, values, and beliefs of specific organizations, but also promotes communication and interaction among members of the organization as well as with internal and external units or groups. This enables the integration of explicit and tacit knowledge within the organization into new knowledge. The leadership behavior of organizational leaders is a decisive factor in the efficiency of organizational knowledge acquisition. High decision-making quality can effectively increase the efficiency of business operations. It is proved that decision-making performance and business-process performance influence business performance (Aydiner et al., 2019). Therefore, decision-making quality is the backbone of the relationship between corporate TMT human capital and sustainable business growth. In light of the above, this study proposes the following hypothesis.

*Hypothesis 2: Decision-making quality fully mediates the relationship between TMT human capital and sustainable business growth.*

### Moderating Effect of Founder Characteristics

The founder of an enterprise is exclusive in terms of human capital. This distinctiveness can be attributed to the unique organizational skills of founders and makes them more likely to have exclusive influence and decision-making power (McClelland, 1961). The founder’s decision-making style and the ability to use resources are critical to the growth rate of the company (Cohen and Wirtz, 2021). Forbes and Milliken (1999) show that the cognitive abilities, intuition, risk tolerance, and vitality of a company’s founders are positively correlated to their decision-making speed. Organizational centralization and formalization are positively and negatively correlated with decision-making speed, respectively. The decision-making speed is quicker in organizations with higher centralization and lower formalization. Moreover, the founders’ cognitive comprehensiveness is positively correlated with the speed of decision-making.

Extant literature shows that the sharing, absorption, and integration of knowledge among individual members of an organization are prerequisites for improving decision-making quality (Ghasemaghaei, 2019). The success of individual knowledge integration depends on the communication efficiency between members. Effective communication can promote the flow and dissemination of different types of knowledge within the organization; founder characteristics are one of the important factors that impact communication efficiency among members. Similarly, other founder characteristics such as rationality in management largely influence the decision-making behavior of corporate TMTs (Liu et al., 2021). An uncompromising organizational decision-making process will lead to a sense of distance, alienation, and even anger among the organization’s members arising from inadequate knowledge sharing and intra-organizational income inequality. In order to resist the state of displeasure and injustice, members of the organization may express their negative reactions through their cognitive behavior. This behavior may be reflected in their negative emotions when facing managers or other members, thereby reducing communication between members of the organization (Khattak et al., 2018). Furthermore, there are differences between members of an organization in terms of interest demands and resource allocation (Baron, 2006). Balancing these conflicts has a significant impact on cooperation between TMT members; practical experience has shown that the organization’s founders play a major role in this process. Research has also suggested that the heterogeneity of TMTs in the service process has an impact on the choice of strategy. TMT’s cognitive diversity can better manage contradiction in business operations (Kanchanabha and Badir, 2021), formulate more reasonable strategies, and maintain consistent growth in business performance (Naranjo-Gil et al., 2008).

In summary, whether it is communication between members of the organization or cooperation between TMT members, the exchange, sharing, and transfer of knowledge and information is crucial (Ghasemaghaei, 2019). Therefore, in business operations, founder characteristics of an enterprise affect the mechanisms between TMT human capital and decision-making quality. Hence, this study proposes the following hypothesis.

*Hypothesis 3: The more open-minded the founder, the higher the decision-making quality on TMT human capital.*

### Integrative Effect of Top Management Team Human Capital and Founder Characteristics

TMT human capital not only effectively mediates the disagreements and contradictions between different interests within an organization, but also enhances the level of trust and collaboration among its members. This provides the foundation to improve decision-making quality in business operations. Thus, TMT human capital significantly improves operational efficiency for businesses. The practical focus of TMTs differs significantly between organizations with different founder characteristics. If the founders are extremely open-minded, TMT human capital mainly focuses on the smooth progress of business operations as well as the acquisition of resources and legal status. Conversely, if the founders are less open-minded, TMTs have to expend considerable effort to resolve communication breakdowns and to address the low efficiency in resource transfer caused by distrust within the organization. This weakens the decision-making quality of corporate TMTs in their business operations (Eisenhardt, 1989). Based on these considerations, this study proposes the following hypothesis.

*Hypothesis 4: The interaction between TMT human capital and founder characteristics promotes sustainable business growth through the mediation of decision-making quality.*

**FIGURE 1:**
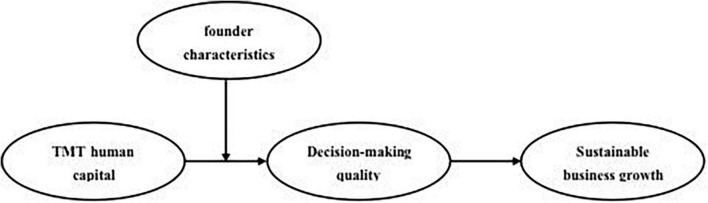
A conceptual model of this study. H1: TMT human capital has a significant and positive impact on sustainable business growth. H2: Decision-making quality fully mediates the relationship between TMT human capital and sustainable business growth. H3: The more open-minded the founder, the higher the decision-making quality on TMT human capital. H4: The interaction between TMT human capital and founder characteristics promotes sustainable business growth through the mediation of decision-making quality.

Combining the above theoretical hypotheses, we construct a theoretical model for this study, as shown in Figure 1. The model reveals the interactions between TMT human capital, founder characteristics, decision-making quality, and sustainable business growth.

## Sample and Methodology

This section describes the sample and methods used in this paper.

### Sample

The sample of this study comprise senior management personnel of private enterprises in Zhejiang Province PRC. And data from the Zhejiang Federation of Enterprises and the Zhejiang Entrepreneurs Association. We randomly distribute 1,000 questionnaires and collect 535 valid responses. Among the 535 responses, male TMT members accounted for 93.8% and the average age of the sample is 37.8 years (*SD* = 8.23).

### Measurements

#### Top Management Team Human Capital

We adopt the measurement scale from Avolio et al. (1999) to measure this variable. Based on participants’ responses, we choose six items. A sample questionnaire item was “*When the TMT (top management team) members work extremely busy, other team members will actively help him to share*.” The various items were evaluated on a five-point Likert scale (1: Strongly disagree; and 5: Strongly agree).

#### Founder Characteristics

We use the seven-item measurement scale developed by Byrne (1999) to measure founder characteristics based on participants’ responses on a five-point Likert scale. A sample questionnaire item was “*The founder is very friendly*.”

#### Decision-Making Quality

We adopt the measurement scale proposed by Tippins and Sohi (2003) to measure decision-making quality based on participants’ responses, and select six items. A sample questionnaire item was “*The strategic decisions made by TMT give the company an advantage over its peers.*” The items were evaluated on a five-point Likert scale.

#### Sustainable Business Growth

We use the seven-item measurement scale compiled by Kropp et al. (2006) to assess sustainable business growth based on participants’ responses. The items were evaluated on a five-point Likert scale. A sample questionnaire item was “*Satisfaction with market share compared to major competitors.*”

#### Control Variables

Carpenter et al. (2004) stated that the characteristics of TMTs might influence organizational behaviors. Furthermore, scholars developed Upper echelon research claimed that the TMTs’ demographic attributes shape the executives’ perceptions, values, and attitudes, which influence the managers’ information-processing and decision-making behaviors (Hambrick and Mason, 1984). Thus, TMT individual characteristics (e.g., age, gender, and years of work experience) were included as control variables in the analysis model of this study for a more accurate investigation of the intrinsic influences among the variables.

### Testing of Questionnaire Reliability and Validity

The purpose of the reliability analysis is to ensure the accuracy and reliability of the questionnaire. Such an analysis reflects the consistency of results obtained by repeated measurements of the same participants using the same method. The level of reliability is usually evaluated with the “α” coefficient. In general, α > 0.7 indicates high questionnaire reliability, 0.6 < α < 0.7 is still within an acceptable range, but α < 0.6 implies that the reliability of the questionnaire is low and warrants revision. This study uses SPSS 20.0 to perform a reliability analysis for each dimension of the questionnaire. The results are presented in Table 1.

**TABLE 1:** Results of reliability analysis.

Variable	Cronbach’s alpha	Items
TMT human capital	0.936	18
Founder characteristics	0.858	4
Decision-making quality	0.807	3
Sustainable business growth	0.943	6

*Cronbach α coefficients of the four variables in this study are all greater than 0.8, thereby confirming the high reliability of the questionnaire used in this study as well as the accuracy and reliability of the sample responses obtained.*

Table 1 shows that the Cronbach α coefficients of the four variables in this study are all greater than 0.8, thereby confirming the high reliability of the questionnaire used in this study as well as the accuracy and reliability of the sample responses obtained. Therefore, further statistical analysis can be conducted.

To analyze the discriminant validity between the four variables, a confirmatory factor analysis (CFA) was performed on these variables using Amos 17.0. The CFA also compares and analyzes the fitting effects of the four-, three-, and single-factor models based on the variables of this study. Results shows that among all models, the four-factor model had the best fit (χ^2^ = 533.33, *p* < 0.01; RMSEA = 0.05, TLI = 0.92, CFI = 0.94, IFI = 0.93), indicating good structural validity between the four variables, as shown in Table 2.

**TABLE 2:** Results of confirmatory factor analysis (CFA).

Model	X^2^	*Df*	RMSEA	TLI	CFI	IFI
Null model^a^	2707.01	351	0.15	0.00	0.00	0.00
Four-factor model	533.33	293	0.05	0.92	0.94	0.93
Three-factor model^b^	740.78	297	0.07	0.78	0.82	0.89
Three-factor model^c^	1014.75	297	0.09	0.64	0.70	0.77
Three-factor model^d^	770.60	297	0.07	0.76	0.80	0.81
Single-factor model^e^	1389.06	299	0.12	0.46	0.54	0.56

**p < 0.01,*p < 0.05.

*^a^In Null model, there is no relationship between all measurement items.*

*^b^Combining TMT human capital and decision-making quality into one potential factor.*

*^c^Combining decision-making quality and sustainable business growth into one potential factor.*

*^d^Combining founder characteristics and decision-making quality into one potential factor.*

*^e^Combining all variables into one potential factor. Results showed that among all models, the four-factor model had the best fit, indicating good structural validity between the four variables.*

## Results and Analysis

### Descriptive Statistics

The descriptive statistics of the empirical data collected in this study are shown in Table 3. As shown in Table 3, there are significant positive correlations between TMT human capital and each of founder characteristics (*r* = 0.13, *p* < 0.05), decision-making quality (*r* = 0.27, *p* < 0.01), and sustainable business growth (*r* = 0.17, *p* < 0.05). In addition, there is a significant positive correlation between founder characteristics and decision-making quality (*r* = 0.19, *p* < 0.01) as well as between decision-making quality and sustainable business growth (*r* = 0.41, *p* < 0.01). Given these results, it can be preliminarily concluded that there are intrinsic interactions between TMT human capital, founder characteristics, decision-making quality, and sustainable business growth.

**TABLE 3:** Summary statistics.

Variable	*M*	*SD*	1	2	3	4	5	6
Age	37.8	8.23	1.00					
Years of work experience	11.60	7.40	0.37**	1.00				
TMT human capital	4.90	0.88	0.07	0.08	1.00			
Founder characteristics	3.86	0.73	0.02	0.03	0.13*	1.00		
Decision-making quality	3.80	0.53	0.06	0.08	0.27**	0.19**	1.00	
Sustainable business growth	3.70	0.74	0.13*	0.11*	0.17*	0.12	0.41**	1.00

**p < 0.01, *p < 0.05.

*Significant positive correlations between TMT human capital and each of founder characteristics, decision-making quality, and sustainable business growth; a significant positive correlation between founder characteristics and decision-making quality; a significant positive correlation between decision-making quality and sustainable business growth.*

### Hypothesis Testing

This study employed a linear regression method to test the proposed research hypotheses. In particular, the study testes the following: the direct effects of TMT human capital on sustainable business growth; the mediating effect of decision-making quality in this relationship; the moderating effect of founder characteristics in the relationship between TMT human capital and decision-making quality; and finally, the mediating effect of decision-making quality between the interaction of TMT human capital and founder characteristics, and sustainable business growth.

#### Direct Effect Testing

This test analyzes the influence of TMT human capital on sustainable business growth. A direct influence relationship model between the two variables was constructed for this purpose. Control variables (age, gender, and years of work experience of corporate TMTs) and the independent variable (TMT human capital) were, in turn, added to the model for analysis. The results are presented in Table 4. Model 6 reveals that TMT human capital has a significant impact on sustainable business growth (β = 0.17, *p* < 0.05); additionally, the model has a significantly good fit (*F* = 6.66, *p* < 0.05). Therefore, H1 is confirmed.

**TABLE 4:** Hypothesis testing.

	Decision-making quality				Sustainable business growth			
	Model 1	Model 2	Model 3	Model 4	Model 5	Model 6	Model 7	Model 8
**Control variables**								
Age	−0.04	−0.0	−0.06	−0.05	−0.02	−0.06	−0.06	−0.06
Gender	0.13	0.08	0.08	0.08	0.12	0.07	0.09	0.07
Work experience	0.06	0.06	0.06	0.07	0.07	0.05	0.09	0.05
**Independent variable**								
TMT human capital		0.27**	0.15*	0.09		0.17*		0.03
**Mediating variable**								
Decision-making quality							0.41**	0.36**
**Moderating variables**								
Founder characteristics			0.17*	0.17*				
TMT human capital * Founder characteristics				0.16*				
*R* ^2^	0.01	0.03	0.06	0.08	0.02	0.12	0.17	0.18
*F*	1.02	6.94*	7.18*	6.98*	1.85	6.66*	7.41*	8.18*
△*R*^2^	0.01	0.03	0.05	0.07	0.01	0.11	0.17	0.18
△F	1.02	6.94*	7.23*	6.25*	1.85	8.97*	14.41**	16.44**

**p < 0.01, *p < 0.05.

*Results of the mediating effect of decision-making quality in the TMT human capital-sustainable business growth relationship. Accordingly, the constructed model included TMT human capital, decision-making quality, and sustainable business growth.*

#### Mediating Effect Testing

This test analyzes the mediating effect of decision-making quality in the TMT human capital-sustainable business growth relationship. Accordingly, the constructed model includes TMT human capital, decision-making quality, and sustainable business growth. And we add control, independent, and mediating variables to the regression equation in turn. Decision-making quality was shown to have a significant positive impact on sustainable business growth (β = 0.41, *p* < 0.01) in Model 7 and a mediating effect on TMT human capital and sustainable business growth (β = 0.03, *p* > 0.05; β = 0.36, *p* < 0.01) based on Model 8. Both Models 7 and 8 were of a good fit (*F* = 7.41, *p* < 0.05 and *F* = 8.18, *p* < 0.05, respectively). Therefore, H2 is confirmed.

#### Moderating Effect Testing

**FIGURE 2:**
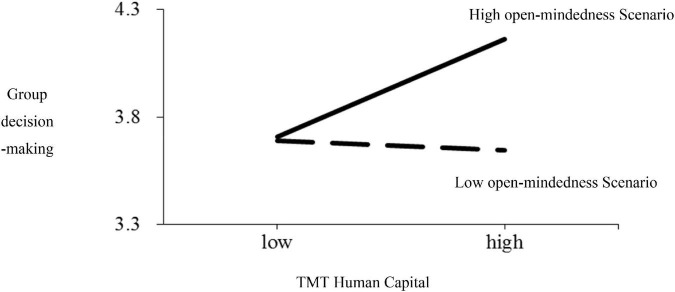
Founder characteristics and TMT human capital-decision-making quality. Founder characteristics had a significant moderating effect on the TMT human capital-decision-making quality relationship. As the open-mindedness of founders increases, the quality of group decision-making increases.

This test is used to analyze the effect of founder characteristics on the relationship between TMT human capital and decision-making quality. Thus, we set decision-making quality as the dependent variable, and add the control, independent, and moderating variables, as well as the products of the independent and moderating variables to the regression equation. To eliminate collinearity, the independent and moderating variables are standardized separately when constructing the product terms. Model 4 in Table 4 shows that founder characteristics have a significant moderating effect on the TMT human capital-decision-making quality relationship (β = 0.16, *p* < 0.05). This implies that the influence of TMT human capital on decision-making quality significantly differs under different founder characteristic scenarios. The more open-minded the founder, the more significant the effect of TMT human capital on decision-making quality. Therefore, H3 is confirmed. This study refers to the method of Cohen et al. (2003) to address the differences between TMT human capital and decision-making quality under different founder characteristic scenarios, which were defined using one standard deviation above and below the mean. The details are shown in Figure 2.

#### Moderated Mediation Effect

This test explores the mediating effect of decision-making quality between the interaction of TMT human capital, founder characteristics, and sustainable business growth. Specifically, this refers to differences in the mediating effect of decision-making quality between the interaction of TMT human capital and sustainable business growth, under different founder characteristic scenarios. This study employees bootstrapping method. Table 5 presents the results, which indicates that the influence of TMT human capital on decision-making quality significantly differs under different founder characteristic scenarios (β = 0.12, *p* < 0.05). In other words, founder characteristics have a significant moderating effect on the influence mechanism between TMT human capital and decision-making quality, thereby further validating H3. The indirect effect displayed in Table 5 further indicates that the impact of TMT human capital on sustainable business growth through decision-making quality is not significant when corporate TMTs have founders with low open-mindedness (β = −0.04, *p* < 0.05). However, if the founders of enterprises have high open-mindedness, TMT human capital is shown to have a significant impact on sustainable business growth through decision-making quality (β = 0.17, *p* < 0.05). There is also a difference in significance levels under the two scenarios (β = 0.13, *p* < 0.05). Therefore, H4 is confirmed.

**TABLE 5:** Moderated mediation effect analysis.

Moderating variables	TMT human capital (X)→ decision-making quality (M)→continuous business growth (Y)				
	Stage		Effect		
	Stage 1	Stage 2	Direct effect	Indirect effect	Total effect
	*P*_*MX*_	*P*_*YM*_	*P*_*YX*_	*P_YM_ P_MX_*	*P_YX_* + *P_YM_ P_MX_*
Low open-mindedness	0.09	0.36**	0.16*	0.04	0.20**
High open-mindedness	0.21**	0.32**	0.05	0.17*	0.22**
Difference	0.12*	−0.04	−0.11	0.13*	0.02

**p < 0.01, *p < 0.05. **P_MX_** represents the impact of TMT human capital on the quality of decision-making quality; **P_YM_** represents the impact of decision-making quality on the sustainable business growth; **P_YX_** represents the impact of TMT human capital on the sustainable business growth. The average High open-mindedness is increased by one standard deviation, and the Low open-mindedness represents the average minus one standard deviation.

## Discussion

(1) In the economic context of China, establishing TMT human capital has a significantly positive impact on business performance. The findings of this study are consistent with those of Smith et al. (1994). They have found that the demographic heterogeneity of TMTs, including age, education level, and tenure, was positively correlated with business performance. In other words, it has a positive effect on business development. Moreover, the overall effectiveness of TMTs reflects the totality of individual capabilities and determines whether a company can grow smoothly.
(2) Existing research indicates that the demographic characteristics (e.g., age, years of working experience), personality traits, and cognitive characteristics of TMTs have an important influence on decision-making quality (Hough and Ogilvie, 2005). However, it also questions why senior management teams make very different decisions even when the above factors are similar. This study shows that founder characteristics are key factors inducing differences in TMT decision-making quality and can be considered to provide a good explanation for such differences.
(3) This study aims to examine whether decision-making quality has a full mediating effect on TMT human capital’s influence on sustainable business growth. The finding that founder characteristics serve as a moderating variable has expanded the scope of research in investigating the intrinsic mechanisms of how TMT human capital influences sustainable business growth. In the business world, TMT human capital is a strength in itself since it influences decision-making quality of organizations based on founder characteristics. This allows senior management to make better strategic decisions and ultimately promote the sustainable growth of companies. Thus, this study enriches research on the intrinsic mechanisms of TMT human capital’s influence on sustainable business growth.
(4) In order to analyze the interaction between the mediating effect of decision-making quality and the moderating effect of founder characteristics, this study uses the bootstrapping method to construct a moderated mediation effect model. The constructed model comprehensively and scientifically evaluates the complex influence relationships between TMT human capital, founder characteristics, decision-making quality, and sustainable business growth in business operations. It also provides a scientific explanation of the influence mechanisms of TMT human capital on sustainable business growth under different founder characteristic scenarios. In summary, this study not only enriches corporate TMT behavioral research in the field of business operations, but also makes a beneficial attempt at using innovative empirical methods.

## Concluding Remarks

In today’s competitive and complex business environment, there is a strong need to acquire and integrate scarce resources in business operations in order to achieve sustainable business growth. Through an empirical investigation, this study explores the moderating and mediating effects of founder characteristics and decision-making quality, respectively, in the relationship between TMT human capital and sustainable business growth. It also discusses the interactive relationships between TMT human capital and each of decision-making quality and sustainable business growth in different organizational backgrounds. The main conclusions are as follows.

Decision-making quality exerts a full mediating effect on the relationship between TMT human capital and sustainable business growth. Usually, TMT human capital meets the operational expectations of all internal members who participate in business operations, by creating a synergy between strong value orientation and capability structures, including remuneration and self-satisfaction. This enables operational efficiency throughout the organization, enhances overall competitiveness, and increases the overall performance of the company’s operational activities. For private enterprises in China, diversity of interest demands, complexity of organizational backgrounds, and imperfection of organizational operations pose significant challenges to the leadership behavior of corporate TMTs. Thus, synergizing TMT value orientation and capability structures in the specific cultural context of China will be challenging. Without sufficient experience in business operations, strengthening decision-making quality would be crucial for corporate TMTs to lead such operations. The TMT leadership behavior in the decision-making process may also stimulate learning awareness among members of the organization. By promoting learning awareness among members, organizations can stimulate the learning desire and motivation of TMTs and, with regard to decision-making quality, focus on a clear direction, create a strong atmosphere for cooperation and coordination, and construct an efficient system to promote sustainable business growth effectively.

Founder characteristics have a significant moderating effect on the influence relationship between TMT human capital and decision-making quality. Specifically, the more open-minded the organization’s founders, the more its TMT can focus on leading business operations and the greater the efficiency of decision-making through TMT value orientation and capability structures. Conversely, the contribution of TMT human capital to the efficiency of decision-making is low. In addition, under different founder characteristic scenarios, there are differences in the manner in which decision-making quality influences TMT human capital’s promotion of sustainable business growth. In order to obtain core knowledge, information, and other resources, TMTs need to build a mutually beneficial relationship between resource owners, business operation participants, and other relevant stakeholders when implementing operational plans. Certain relationships rely on the equal exchange of interests or values, such as resource gathering, political support, and psychological dependence. When an organization is able to facilitate the exchange of knowledge, information, and other resources between corporate TMTs and other relevant stakeholders, it significantly promotes the efficiency of acquiring the necessary knowledge and skills to enhance decision-making quality. Therefore, creating a relatively relaxed organizational environment in business operations is conducive to improving the decision-making quality of TMTs.

## Data Availability Statement

The data that support the findings of this study are available from the corresponding author upon reasonable request.

## Ethics Statement

The studies involving human participants were reviewed and approved by the Tongji University. The participants provided their written informed consent to participate in this study. Written informed consent was obtained from the individuals for the publication of any potentially identifiable images or data included in this article.

## Author Contributions

FH: conceptualization (lead), writing—original draft (lead), formal analysis (lead), and writing—review and editing (equal). QZ: software (lead) and writing—review and editing (equal). YS: methodology (lead) and writing—review and editing (equal). XL: conceptualization (supporting), writing—original draft (supporting), and writing—review and editing (equal).

## Conflict of Interest

The authors declare that the research was conducted in the absence of any commercial or financial relationships that could be construed as a potential conflict of interest.

## Publisher’s Note

All claims expressed in this article are solely those of the authors and do not necessarily represent those of their affiliated organizations, or those of the publisher, the editors and the reviewers. Any product that may be evaluated in this article, or claim that may be made by its manufacturer, is not guaranteed or endorsed by the publisher.
