# Supplementary material for: The Influence of Top Management Team Human Capital on Sustainable Business Growth
Source: Front Psychol. 2021 Nov 25;12:773689. doi: 10.3389/fpsyg.2021.773689 (PMC8660561; doi:10.3389/fpsyg.2021.773689)
Supplement: Supplementary file 1 [file Data_Sheet_1.pdf]

TMT human capital

----Value Orientation (five-point Likert scale,1 means completely inconsistent and 5 means completely consistent**)**

1. When the TMT (top management team) members work extremely busy, other team members will actively help him to share.
2. Each TMT member is willing to adjust their responsibilities for the team to work smoothly.
3. TMT members are willing to help each other with tasks.
4. I will not harm the interests of the organization for my own benefit.
5. I have a consistent attitude toward my superiors and subordinates.
6. I keep my word and keep my promise.
7. I don't pass the buck when things go wrong at work but try to improve.
8. I am responsible, loyal and dedicated to my work.
9. I feel responsible for maximizing the long-term return of the company.

----TMT Competency Structure (five-point Likert scale,1 means completely inconsistent and 5 means completely consistent**)**

1. I can also discover new business opportunities in areas I have never been involved in.
2. Discovering business opportunities usually requires years of experience in a particular industry or region.
3. I am particularly sensitive to discovering new business opportunities.
4. I always think about developing business in new ways.
5. I am good at combining ideas, opinions and observations from various sources.
6. I like trying to change things in different ways.
7. I have a clear learning goal and always take the initiative to learn the latest knowledge.
8. I can make full use of various channels to search and absorb useful information for myself.
9. I can timely summarize and absorb the experience and lessons of myself and others.
10. I can apply what I have learned in management practice.
11. I can write documents concisely, quickly, and easily understood.
12. I can make full preparation before talking with people and explain business problems directly.
13. I can interact with the rapper and express my focus in a clear and concise manner.
14. I can develop long-term trust relationships with stakeholders in the process of cooperation.
15. I can build relationships with potential stakeholders.
16. I often put a lot of energy and resources into developing relationships with stakeholders.
17. I can build a highly effective management team according to the needs of my work.
18. I can effectively combine the limited resources of the enterprise to maximize the effectiveness of the limited resources of the enterprise.

Founder Characteristics (five-point Likert scale,1 means completely inconsistent and 5 means completely consistent)

1. The founder is very friendly.

2. We feel very proud to follow the founder.

3. We are very confident in the founder.

4. The founder is not suspicious and willing to trust his subordinates.

5. The founder can present the prospect of the enterprise to his subordinates.

6. The founder can guide everyone to find the meaning of hard work.

7. The founder can encourage us to use new methods to solve problems in our work.

8. The founder can encourage us to challenge what we used to think was right.

9. The founder can emphasize to everybody should think actively and use wisdom to solve the problem that the enterprise faces.

10. The founder can support his subordinates to keep learning, self-improvement and development.

11. The founder can understand the subordinates' psychological feelings and ideas, and care about the subordinates' difficulties.

12. The founder is willing to meet the requirements of the subordinates if they can meet his requirements.

13. The founder can give corresponding reward according to subordinate work performance.

14. The founder will give corresponding rewards to different talents.

15. The founder will try not to change the status quo if the performance of subordinates and performance standards.

16. The founder will point it out and ask for improvement if the work of his subordinates is not up to standard.

17. The founder can ask subordinate must accord with what regulation and achieve what level.

18. The founder asked me to fully obey his leadership.

19. The founder gives me a lot of work pressure.

20. The meeting will make the final decision according to the founder's will.

21. The founder cares about my personal life and daily life.

22. The founder will meet the requirements according to my personal needs.

23. The founder is a decent person and won't cheat others.

24. The founder treats us impartially

25. The founder can lead by example

Decision-making quality (five-point Likert scale,1 means completely inconsistent and 5 means completely consistent)

1. Every strategic decision made by TMT can usually promote the development of the enterprise in a good direction.

2. The strategic decisions made by TMT give the company an advantage over its peers.

3. The strategy made by TMT makes the business operation, development scale and prospects are very good.

4. The identification cost of decisions made by TMT is high.

5. The communication cost of decisions made by TMT is high.

6. The time cost of decisions made by TMT is high.

7. The conflict cost among TMT members is high.

8. TMT is quick to respond to environmental changes.

9. TMT spends less time making major decisions.

Business Growth (five-point Likert scale,1 means very dissatisfied and 5 means very satisfied)

1. Satisfaction with the growth rate of sales revenue compared to major competitors.
2. Satisfaction with net profit margins compared to major competitors.
3. Satisfaction with return on investment compared to major competitors.
4. Satisfaction with corporate solvency compared to major competitors.
5. Satisfaction with business capabilities compared to major competitors.
6. Satisfaction with market share compared to major competitors.
7. Satisfaction with the quality of the product/service/project compared to major competitors.
8. Satisfaction with the development of new products/services/projects compared to major competitors.
9. Satisfaction with company to absorb and retain key employees compared to major competitors.
10. Customer satisfaction with the business compared to major competitors.
11. Employee satisfaction with the company compared to major competitors.
12. Satisfaction of other stakeholders compared to major competitors.
